# Imaging vocal fold medial surface vibration across chest, head, whistle, and creaky voice production using optical coherence tomography: A single subject study

**DOI:** 10.1121/10.0044145

**Published:** 2026-06-09

**Authors:** Zhaoyan Zhang, Anna Wisniowiecki, Melissa Treinkman, Bette Wo, Emma Glenane, Diego E. Razura, M. Eugenia Castro, Brian E. Applegate, Michael M. Johns

**Affiliations:** 1 Department of Head and Neck Surgery, University of California, Los Angeles, Los Angeles, California 90095, USA; 2 Department of Otolaryngology Head and Neck Surgery, USC Caruso, 1537 Norfolk Street, Los Angeles, California 90033, USA; 3 Department of Biomedical Engineering, Texas A&M University, College Station, Texas 77843, USA; 4 School of Dramatic Arts, University of Southern California, Los Angeles, California 90089, USA; 5 Department of Communication Disorders, California State University, Los Angeles, Los Angeles, California 90032, USA; 6 Alfred E. Mann Department of Biomedical Engineering, University of Southern California, Los Angeles, California 90089, USA; 7 Ming Hsieh Department of Electrical and Computer Engineering, University of Southern California, 1042 Downey Way, Los Angeles, California 90089, USA

## Abstract

This single-subject study used optical coherence tomography to image medial surface vibratory patterns during the production of chest, head, whistle, and creaky voice. Chest and head registers differed in medial surface thickness, with the chest voice produced with thicker vocal folds, greater vibratory amplitude, and a larger vertical phase difference than the head voice and the other voice qualities. The whistle voice was produced with tightly approximated vocal folds that were thicker than in the head voice and showed small but measurable vibrations. An alternating creaky and regular voice was produced without observable changes in vocal fold thickness.

## 1. Introduction

Vocal registers are often described in terms of perceptual evaluation, acoustics, aerodynamics, and imaging of vocal fold vibration from a superior, endoscopic view. Physiologically, the vocal fold medial surface shape, particularly the vertical thickness, has long been hypothesized to play an important role in regulating vocal registers. Based on x-ray tracings of a human subject producing chest and falsetto voices, van den Berg (1968) argued that chest voices are often produced with thick vocal folds, whereas falsetto voices are often produced with thin vocal folds. This hypothesis was supported by recent computational studies (Zhang, 2025), which showed that the medial surface vertical thickness plays a dominant role in determining the glottal closure pattern and the voice source spectral shape, two important parameters that differentiate different registers. However, due to difficulties in imaging the medial surface, this hypothesis has yet to be directly verified in live humans. The goal of this study was to test this hypothesis in a human subject by imaging and comparing the medial surface vibration pattern across different vocal registers and voice qualities, including chest, head, creak, and whistle.

Due to limited access to the larynx, imaging the medial surface shape and vibration is challenging in live humans. Early imaging studies in live humans used x rays to image vocal fold shape in the coronal plane (e.g., Hollien, 1962; van den Berg, 1968) at different frequencies of voice production. Due to the long imaging time required in these experiments, these studies were only able to measure the vocal fold shape averaged over many cycles of vocal fold vibration. The first imaging study of vocal fold vibration in the coronal plane in live humans was probably by Hollien *et al.* (1968) using stroboscopic laminagraphy. Other imaging modalities have also been used in recent studies, including, for example, ultrasound (Tsai *et al.*, 2009; Jing *et al.*, 2018), optical coherence tomography (OCT) (Kaiser *et al.*, 2009; Coughlan *et al.*, 2016; Sharma *et al.*, 2021), and dynamic magnetic resonance imaging (Fischer *et al.*, 2026). These studies often focused more on methodology development rather than the differences in medial surface dynamics across different voice conditions. The potential differences in medial surface shape and vibration patterns between registers have yet to be systematically investigated in live humans.

In this single-subject study, we imaged vocal fold vibratory patterns in the coronal plane across different vocal registers and voice qualities using OCT. OCT is a depth-resolved technology that allows visualization of tissue microstructures and movement within the vocal folds. This makes it possible to image the vocal fold medial surface vibration, even when it is hidden from a superior endoscopic view. In this study, we applied OCT to image and compare vocal fold vibratory patterns in the coronal plane across different vocal registers and voice qualities. Our findings support previous hypotheses regarding the differences between chest and head registers in vocal fold thickness and vocal fold vibratory pattern. Our results also provide new insights into the vocal fold configuration in the production of creaky and whistle voice.

## 2. Methods

**Fig. 1.**
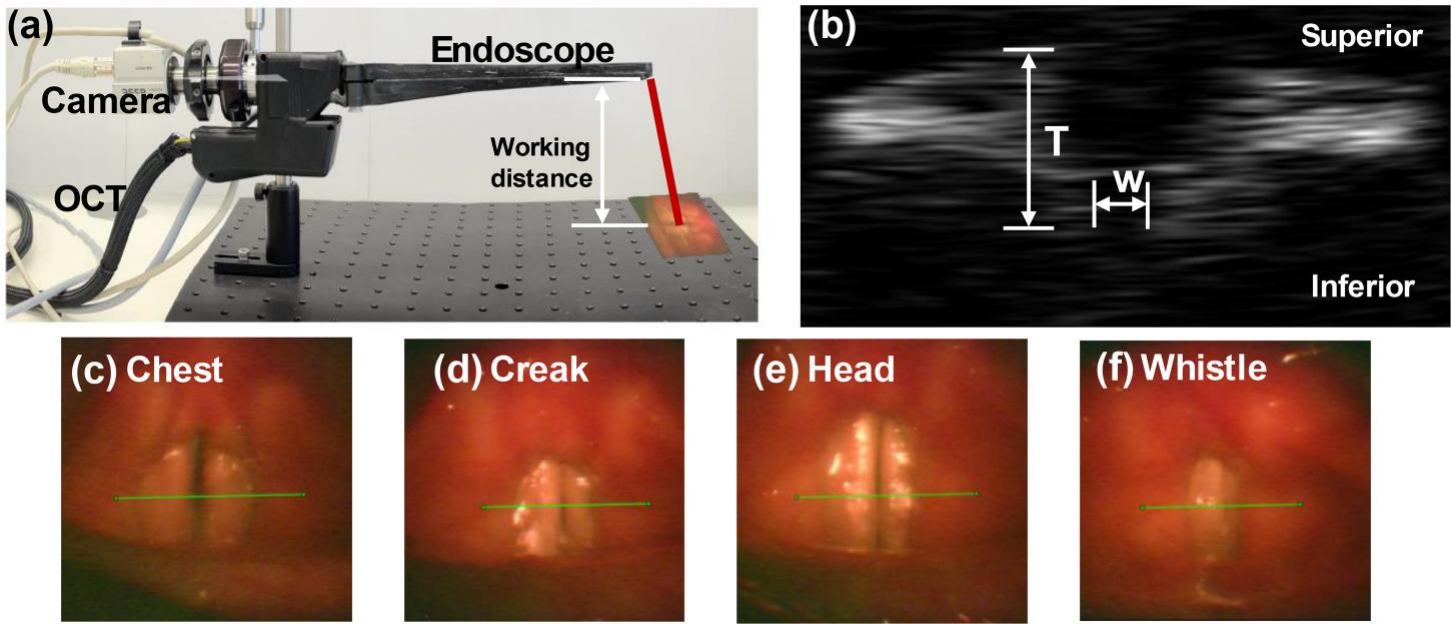
(a) The OCT system. (b) Measurement of vocal fold vertical thickness *T* and glottal width *w* in the coronal OCT image. (c)–(f) Selected endoscopic images of the vocal folds, where the green line indicates the plane of OCT imaging. The images were scaled to account for differences in working distance, allowing comparison across conditions.

A female singer in her early 40s with more than 25 years of training and professional operatic singing experience participated in this study. The subject was instructed to produce and sustain a forward and bright vowel /i/ in the chest and head registers (Table 1). The subject also produced a high-frequency irregular phonation (referred to as creak in this study) and a very high-frequency phonation as often described in Western classical pedagogy as a “whistle register” (Herbst, 2020). Although the vowel /i/ was intended, the subject produced /e/ for the chest and creak voice. For each condition, vocal fold vibration in the mid-coronal plane was imaged using a transoral rigid laryngoscope integrated with an OCT system [Fig. 1(a)] (Wisniowiecki *et al.*, 2023). The OCT system was based on a Mach–Zehnder interferometer using a swept-source laser (Insight, Inc., Broomfield, CO; λ_0_ = 1.304 μm). Using a fast-scanning micro-electromechanical mirror, we were able to achieve B-scan (cross-sectional scanning) rates of 1020 Hz, with a resolution of 22.6 μm in the superior-inferior direction and a resolution of ∼250 μm in the medial-lateral direction at a working distance of 70 mm. We used a variable optical delay line to enable a variable working distance range of 54–110 mm from the tip of the laryngoscope. For each voice task, multiple OCT data collections were made, with each data collection 1-s long. The produced speech sound and electroglottographic (EGG) data were also recorded synchronously using a B&K 1/2-inch microphone (28 cm from the lips; Brüel & Kjaer, Naerum, Denmark) and a two-channel electroglottography (Glottal Enterprises, Syracuse, NY), respectively. The recorded sound for the four voice qualities is included in Mm. 1. A co-aligned video camera also captured standard endoscopic video simultaneously with acquired OCT scans.

**Table 1.** Voice conditions, fundamental frequency F0, and vibratory measures extracted from OCT. For creak, measures with an asterisk (*) were extracted from the regular phonation segment during the same production. Note that the F0 in whistle was higher than the OCT frame rate of 1020 Hz; however, the reconstruction method used to analyze the OCT data is not limited by the frame rate.

	F0 (Hz)	Thickness (mm)	Vibration amplitude (mm)	Closed quotient
Chest	218	2.05	0.49	0.47
Creak	334	1.01	0.28^*^	0.36^*^
Head	697	0.87	0.16	0.29
Whistle	1288	1.22	n/a	1

**Mm. 1.** The recorded audio in the order of chest, head, whistle, and creak.

For all conditions except creak, a novel algorithm based on retrospective gating was implemented in matlab (version R2022a; MathWorks, Natick, MA) to create temporally and spatially resolved movies of the *in vivo* vocal fold vibratory cycle from the acquired B-scans (Wisniowiecki *et al.*, 2026; Razura *et al.*, 2026). OCT data in a single cross-sectional plane were collected over time during phonation to acquire image sequences representing vocal fold vibration within a coronal plane. The OCT images were further reconstructed into a sequence of images representing phase-resolved vocal fold vibration within one vocal fold vibratory cycle. Relative phase was assigned to each B-scan and A-line (one-dimensional vertical scans) according to the detected phonation frequency and the OCT scan and laser sweep rates. A set of adjacent B-scans were then assigned to represent phases of a single glottal cycle and reordered based on the relative glottal phase of the first A-line acquired in each B-scan. The reordered B-scan set was interpolated across phase using the maximum and minimum measured phases in each B-scan set to create an equally spaced interpolant with 100 phase steps over 2π. Interpolation was performed for each lateral position using the A-line-specific phase to create single-phase images representing each time point over the glottal cycle. For the creaky voice, which is inherently irregular, no phase reconstruction was performed.

For each condition, the medial surface vertical thickness was measured as the vertical span of the medial surface at the beginning of the closing phase of vocal fold vibration [Fig. 1(b)], where the inferior margin of the medial surface can be observed. The closed quotient (CQ) of vocal fold vibration was measured as the ratio between the number of frames where the two vocal folds were in contact over the total number of frames in one oscillation cycle. The vocal fold vibration amplitude was measured as the difference between the maximum and minimum glottal width [Fig. 1(b)] divided by two.

## 3. Results

### 3.1 Chest voice

**Fig. 2.**
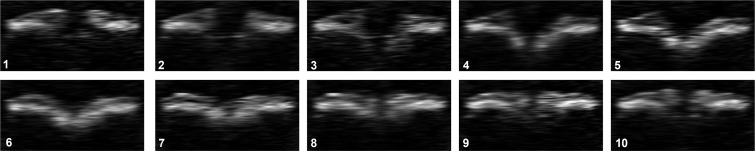
Chest voice. Mid-coronal images of the vocal fold in ten equally spaced instants within one oscillation cycle. Also see Mm. 2 where units are available.

Figure 2 shows the coronal images of the vocal folds in ten equally spaced instants within one vibratory cycle during a chest voice production. A video of vocal fold vibration can be found in Mm. 2. The vocal folds had a vertical thickness of 2.05 mm along the medial surface and vibrated with a relatively large vibration amplitude of 0.49 mm. The vibration led to vocal fold contact for a considerable portion of the vibratory cycle, with a CQ of about 0.47. Vocal fold contact started inferiorly on the medial surface and propagated upwards, creating a large vertical phase difference in medial-lateral movement between the lower and upper margins of the medial surface. This vertical phase difference led to a convergent glottal channel during the opening phase and a divergent glottal channel during the closing phase. The vocal folds also exhibited noticeable vertical movement, with the vocal folds moving upward during the opening phase and downward during the closing phase. This vibratory pattern is similar to observations in excised larynx experiments (e.g., Baer, 1975; Döllinger and Berry, 2006) and computational simulations (Zhang, 2016, 2025).

**Mm. 2.** Medial surface vibration in the coronal plane during the chest voice. As vertical distance in OCT depends on refractive index of the medium, axial measurements in the vertical direction are scaled according to the refractive index of the medium in which the measurement is made (i.e. in soft tissue, n = 1.4, while in air, n = 1). Therefore, vertical image dimensions are provided for both media. Lateral distance is defined by acquisition geometry and independent of refractive index.

### 3.2 Head voice

**Fig. 3.**
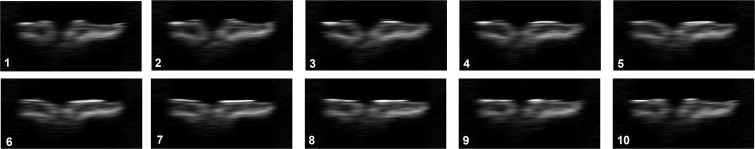
Head voice. Mid-coronal images of the vocal fold in ten equally spaced instants within one oscillation cycle. Also see Mm. 3.

Figure 3 shows the coronal images of medial surface vibration for a head voice production, a video of which is included as Mm. 3. Compared with the chest voice, the vocal folds were much thinner, with a vertical thickness of 0.87 mm, almost half as in the chest voice. The medial-lateral vibration amplitude was also much reduced (0.16 mm in head vs 0.49 mm in chest). Vocal fold contact was still observed but was brief (with a CQ of 0.29) and localized, unlike the chest voice in which the point of contact continuously propagated upwards along the entire medial surface. While a wave motion can be observed along the medial surface, its wave amplitude was much smaller. As a result, although the glottal channel shape did change over time, it did not exhibit the clear alternating pattern between convergent and divergent shapes as observed in the chest voice.

**Mm. 3.** Medial surface vibration in the coronal plane during the head voice.

### 3.3 Whistle voice

The whistle voice production exhibited subtle vocal fold movement in the coronal plane that is difficult to see in still images in individual frames (thus none shown) but can be clearly observed in the video Mm. 4. The two vocal folds were in contact for the entire cycle of oscillation, indicating strong vocal fold approximation. As a result, it was difficult to identify the medial surface of each fold or to calculate the vibration amplitude. Despite the very high frequency (1288 Hz), the vertical thickness was estimated to be about 1.22 mm, smaller than that in the chest but larger than in the head, indicating that the subject was simultaneously activating the thyroarytenoid muscle to maintain strong adduction at this high frequency. The strong adduction was also confirmed from the endoscopic images, which showed that the entire visible glottis was completely closed along the anterior-posterior direction [Fig. 1(f)].

The vibration amplitude in the whistle voice was the smallest of all conditions. An up-and-down motion of the vocal folds can be observed, and a wave motion can be vaguely observed on the superior surface, particularly on the left vocal fold (the fold shown on the right in Mm. 4). Spectral analysis of the voxel intensity near the medial surface showed a clear peak at the whistle frequency, although with a reduced signal-to-noise ratio (SNR) of 2.5 compared with the chest voice images (SNR > 4), likely due to the low vibration amplitude. Spatial mapping of the voxel spectral peaks revealed that oscillations at the whistle voice frequency were localized to the vocal fold region in the images, indicating the observed movement was related to the whistle voice production rather than an artifact. These observations indicate that vocal folds were indeed vibrating during the whistle voice production, although the amplitude was very small. This was also confirmed in the simultaneously collected EGG data, which showed a small amplitude but clear oscillation at the fundamental frequency of the whistle voice. However, this vibration was too small to be observed in the endoscopic video.

**Mm. 4.** Medial surface vibration in the coronal plane during the whistle voice.

### 3.4 Creaky voice

**Fig. 4.**
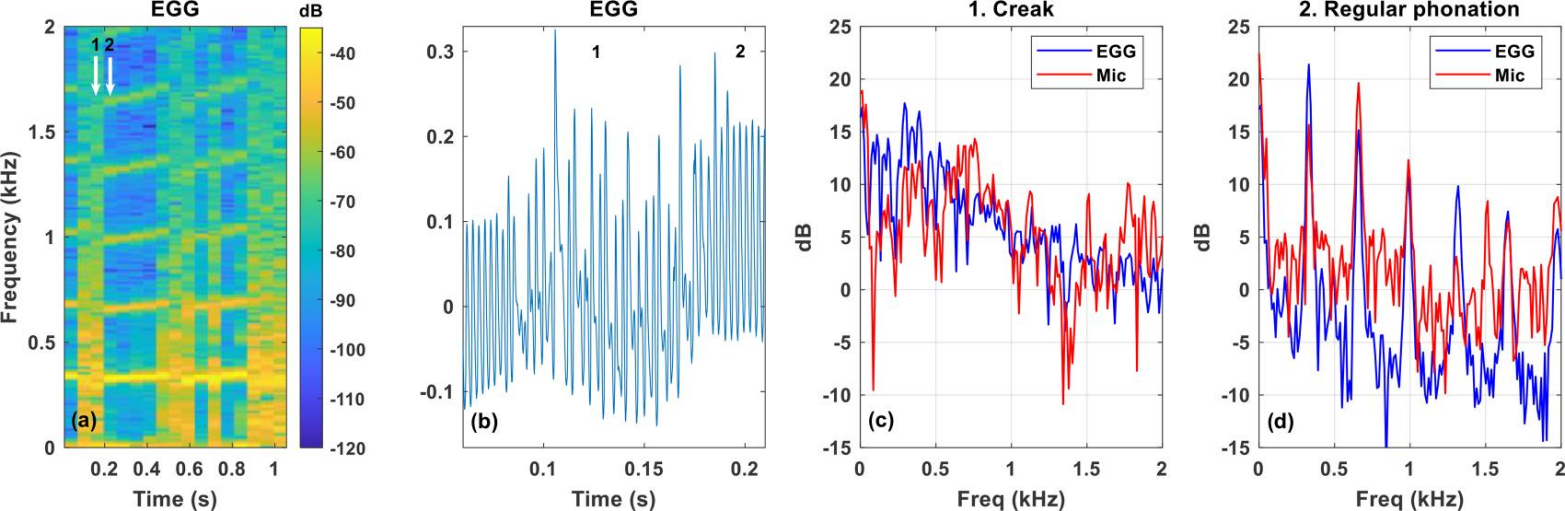
(a) Spectrogram and (b) time series waveform of the EGG data for the creaky voice condition, and the EGG and microphone spectra during a segment of (c) creaky voice and (d) regular phonation, which are labeled 1 and 2, respectively.

The subject also produced a creaky voice around 334 Hz. The spectrogram of the EGG data for the duration of OCT data collection is shown in Fig. 4(a). The production alternated between segments of irregular phonation (the creaky voice) and regular phonation, examples of which are labeled 1 and 2 in Fig. 4, respectively. The corresponding time series waveform of the EGG data for these two segments is also shown in Fig. 4(b). The spectra of the EGG and microphone data for these two segments are shown in Figs. 4(c) and 4(d). The creaky segment had an irregular EGG waveform with time-varying periods [middle portion in Fig. 4(b)], and the spectra [Fig. 4(c)] show many peaks with a frequency spacing ranging between 50 and 80 Hz. In contrast, for the regular phonation [Fig. 4(d)], the EGG and microphone spectra show a clear harmonic structure with a fundamental frequency of 334 Hz. The difference in H1-H2 (amplitude difference between the first and second harmonics) between the EGG and microphone data were large (about a 10-dB decrease), indicating that the second harmonic was likely close in frequency to the first vocal tract resonance during the regular phonation. While vocal instability may occur as the first vocal tract resonance approaches a source harmonic, whether this was the case in the creaky segment cannot be determined from the data collected in this study.

Due to the irregular nature of the creaky voice, OCT phase reconstruction was not performed on the creaky segment of the voice. Instead, the original OCT images are included in Mm. 5, where the creaky segment occurs between about 6–27 s into the video. The images show that the vocal folds were moderately approximated, with a vertical thickness of about 1.01 mm. This indicates a vocal fold posture that lies between the chest and head voices, which may be related to its intermediate fundamental frequency (Table 1). There was no noticeable difference in thickness between the creaky and regular segments, indicating subtle laryngeal adjustments, if anything at all. Again, it is difficult to determine whether the vocal instability was due to laryngeal adjustments or source-filter interaction. Further quantitative analysis of the vibratory pattern was difficult due to the limited frame rate of the OCT.

**Mm. 5.** Medial surface vibration in the coronal plane during the creaky voice production (without reconstruction).

Vocal fold vibration during the segment of regular phonation was reconstructed to provide further insight into the vocal fold configuration. The video is included in Mm. 6. During the regular phonation, the vocal fold vibration amplitude was 0.28, slightly larger than the head voice but smaller than the chest voice. An alternatingly convergent and divergent glottal channel shape can be observed, indicating a wave motion that was stronger than in the head voice but weaker than the chest voice. Vocal fold contact can be observed, with a CQ of 0.36, higher than the head voice.

**Mm. 6.** Medial surface vibration in the coronal plane during the regular phonation segment of the creak condition.

## 4. Discussion and conclusion

In this study, we reported, for the first time, medial surface vibratory patterns across different vocal registers and voice qualities in a human subject. Our results support van den Berg's hypothesis (van den Berg, 1968) that register changes involve adjustments in the medial surface vertical thickness, with the chest voice produced with thick vocal folds and the higher voice qualities (head, creak, and whistle) produced with thin vocal folds. Consistent with the predictions of our simulation studies (Zhang, 2016, 2025), the thick vocal folds in the chest voice vibrated with a long duration of glottal closure, a large vibration amplitude, and an alternatingly convergent and divergent glottal channel shape in the coronal plane. In contrast, the thin vocal folds in the head and creaky voices vibrated with a reduced duration of glottal closure, reduced vibration amplitude, and smaller change in the glottal channel geometry. In general, both the CQ and vibration amplitude increased with medial surface vertical thickness.

Different mechanisms have been proposed in the literature for the production of the high-frequency whistle voice (Herzel and Reuter, 1997; Tsai *et al.*, 2008; Herbst, 2020; Wingerath and Grawunder, 2023), including a mechanism involving flow-induced vocal fold vibration similar to that in the chest voice and an aeroacoustic mechanism involving glottal or supraglottal flow instabilities. While vocal fold vibration was observed in the whistle voice of this study, the vocal folds were in contact during the entire production, and we did not observe periodic glottal opening and closing as reported in Echternach *et al.* (2013) and Echternach *et al.* (2024). This seems to suggest potentially different production mechanisms across these studies, despite similar pitch range. On the other hand, the presence of vocal fold vibration does not necessarily rule out the possibility that some aeroacoustic mechanisms play a role in the whistle production. At this high frequency, the observed small-amplitude vocal fold vibration may very well be excited by some flow instabilities. Further investigations are required to clarify these questions.

Despite the very high frequency, the whistle voice in our study was produced with a thickness larger than in the head voice, at least at the mid-membranous location where the OCT images were obtained, and the vocal folds were tightly approximated. This indicates strong engagement of the adductory muscles. Such strong adductory muscle activity often limits the amount of stiffness and tension increase that can be achieved by the cricothyroid muscle, thus seemingly interfering with the goal of reaching the very high notes. It is possible that such strong adduction may suppress vibration in some part of the membranous vocal folds, thus reducing the effective vibrating length of the vocal folds and facilitating a very high-frequency production. Alternatively, if an aeroacoustic mechanism is involved, such adduction may be required to prevent the airflow from transitioning to turbulence, a condition that is essential to tonal sound production by jet flow (Zhang *et al.*, 2004).

Our study demonstrated the potential of OCT in observing and monitoring the three-dimensional vocal fold vibration, particularly in the vertical dimension. This opens opportunities to better understand vocal fold control mechanisms underlying different voice qualities and vocal techniques. A main limitation of the OCT is the limited penetration depth into the vocal folds, which is about 1–2 mm, depending on OCT wavelength and tissue optical properties. Thus, it may not allow visualization of the entire vocal fold thickness, particularly when the vocal folds are tightly adducted. For the same reason, the thickness measures of this study may have been underestimated. Another limitation is the frame rate, which is 1020 Hz, which does not provide sufficient temporal resolution when imaging irregular vocal fold vibration at high frequencies, as in the case for the creaky voice in this study.

## Data Availability

The data that support the findings of this study are available from the corresponding author upon reasonable request.
